# A phase II trial of sitravatinib + nivolumab after progression on immune checkpoint inhibitor in patients with metastatic clear cell RCC

**DOI:** 10.1093/oncolo/oyaf053

**Published:** 2025-04-11

**Authors:** Andrew W Hahn, Nabil Adra, Ulka Vaishampayan, Lianchun Xiao, Nazli Dizman, Ying Yuan, Sagar S Mukhida, Matthew T Campbell, Jianjun Gao, Amado J Zurita, Eric Jonasch, Nizar M Tannir, Amishi Y Shah, Pavlos Msaouel

**Affiliations:** Department of Genitourinary Medical Oncology, The University of Texas MD Anderson Cancer Center, Houston, TX 77030, United States; Division of Hematology/Oncology, Department of Internal Medicine, Indiana University, Indianapolis, IN 46202, United States; Division of Hematology/Oncology, Department of Internal Medicine, University of Michigan, Ann Arbor, MI 48109,United States; Department of Biostatistics, The University of Texas MD Anderson Cancer Center, Houston, TX 77030, United States; Department of Genitourinary Medical Oncology, The University of Texas MD Anderson Cancer Center, Houston, TX 77030, United States; Department of Biostatistics, The University of Texas MD Anderson Cancer Center, Houston, TX 77030, United States; Department of Genitourinary Medical Oncology, The University of Texas MD Anderson Cancer Center, Houston, TX 77030, United States; Department of Genitourinary Medical Oncology, The University of Texas MD Anderson Cancer Center, Houston, TX 77030, United States; Department of Genitourinary Medical Oncology, The University of Texas MD Anderson Cancer Center, Houston, TX 77030, United States; Department of Genitourinary Medical Oncology, The University of Texas MD Anderson Cancer Center, Houston, TX 77030, United States; Department of Genitourinary Medical Oncology, The University of Texas MD Anderson Cancer Center, Houston, TX 77030, United States; Department of Genitourinary Medical Oncology, The University of Texas MD Anderson Cancer Center, Houston, TX 77030, United States; Department of Genitourinary Medical Oncology, The University of Texas MD Anderson Cancer Center, Houston, TX 77030, United States; Department of Genitourinary Medical Oncology, The University of Texas MD Anderson Cancer Center, Houston, TX 77030, United States

**Keywords:** MET inhibitor, TAM, targeted therapy, immunotherapy rechallenge

## Abstract

**Background:**

Sitravatinib, an oral multi-kinase inhibitor targeting VEGFR, TAM, and MET, has been shown to resensitize the tumor microenvironment to immune checkpoint inhibitors (ICI) by reducing immune-suppressive myeloid cells in metastatic clear cell RCC (ccRCC). ICI is the standard first-line (1L) treatment of metastatic ccRCC, and there is unmet need for improved treatment outcomes after progression on ICI. We hypothesized that sitravatinib plus nivolumab would revert an immunosuppressive tumor microenvironment (TME) to improve clinical outcomes.

**Methods:**

In this investigator-initiated, phase II, multicenter trial (NCT04904302), patients with progressive metastatic ccRCC after 1-2 lines of treatment were enrolled into 3 cohorts: (1) 1L nivolumab + ipilimumab, (2) 1L pembrolizumab + axitinib, (3) prior cabozantinib or lenvatinib and ICI. Starting dose of sitravatinib was 100 mg PO daily and nivolumab was 480 mg IV every 4 weeks. The co-primary endpoints were objective response rate (ORR) and disease control rate (DCR) at 24 weeks. The study was designed to enroll 88 patients with an interim analysis for futility in each cohort using a BOP2 design, but it was terminated early due to discontinuation of sitravatinib development.

**Results:**

Fourteen patients were enrolled with 2 in cohort A, 6 in cohort B, and 6 in cohort C. Across all cohorts, the ORR was 15.4% (2/13, 1 not evaluable) and DCR at 24 weeks was 35.7% (5/14). DCR at 24 months was 63% for Cohort A + B and 0% for Cohort C. Median progression free survival was 5.5 mo [95% CI 3.8—not reached (NR)], and median overall survival was 13.3 mo (95% CI 8.77—NR). Six patients (42.9%) experienced a grade 3-4 adverse event (AE) and 2 patients (14.3%) experienced an immune-mediated AE.

**Conclusion:**

In this small phase 2 trial with limited sample size due to early termination, sitravatinib plus nivolumab demonstrated a manageable safety profile and produced modest clinical benefit. The observed responses occurred in patients who did not receive prior treatment with cabozantinib or lenvatinib. (ClinicalTrials.gov Identifier: NCT04904302).

**Lessons learned**

In this phase II trial with limited sample size due to early termination, sitravatinib plus nivolumab demonstrated a manageable safety profile and produced modest clinical benefit in patients with metastatic clear cell RCC whose cancer progressed on an immune checkpoint inhibitor and did not previously receive cabozantinib or lenvatinib.

## Discussion

Immune checkpoint inhibitors (ICI) that target PD-1 are the backbone of first-line treatment for patients with metastatic clear cell renal cell carcinoma (ccRCC). While ICIs have improved outcomes, there is an unmet need to improve response to subsequent therapies. Sitravatinib is an oral multi-kinase inhibitor targeting the VEGFR, TAM, and MET receptors. In samples from patients who received sitravatinib + nivolumab after their cancer progressed on an antiangiogenic targeted therapy, sitravatinib produced a proinflammatory state by decreasing immune-suppresive myeloid cells in the peripheral blood and increasing the T-cell to immune-suppresive myeloid cell ratio in the tumor. In the present study, we hypothesized that sitravatinib plus nivolumab would revert an immunosuppressive tumor microenvironment after progression on ICI to improve clinical outcomes.

**Figure 1.**
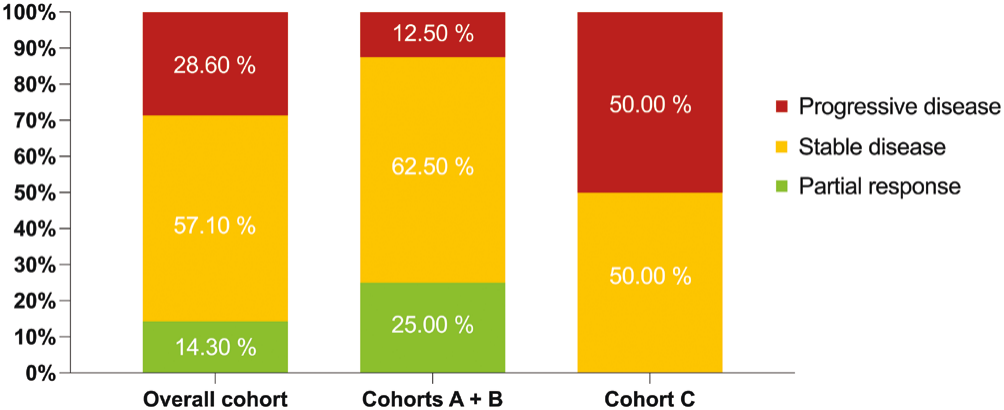
RECIST version 1.1 response rates for the overall cohort, cohorts A + B, and cohort C. Cohort A is progression on first-line nivolumab + ipilimumab, cohort B is progression on first-line pembrolizumab + axitinib or second-line anti-PD-1 therapy after receiving 1L VEGF-targeted monotherapy, and Cohort C progression on first-line or second-line cabozantinib or lenvatinib ± everolimus either before, after, or in combination with anti-PD-1 ICI

Sitravatinib and Nivolumab After Prior Immunotherapy (SNAPI) was a multicenter, single-arm, open label, phase II clinical trial that enrolled patients into 1 of 3 cohorts based upon prior treatment. Fourteen patients enrolled prior to study termination. In the overall cohort, ORR was 14.3% and DCR at 24 weeks was 35.7%. Since sitravatinib targets a similar spectrum of RTKs to cabozantinib and lenvatinib, we analyzed ORR and DCR for cohorts A plus B (no prior cabozantinib or lenvatinib) and cohort C (prior cabozantinib or lenvatinib). We found that the ORR was 25% and the DCR at 24 weeks was 62.5% in cohorts A plus B, whereas, the ORR was 0% and the DCR at 24 weeks was 0% in cohort C (**Figure 1**).

Prior to study termination, sitravatinib plus nivolumab demonstrated a manageable safety profile and produced modest clinical benefit in patients who did not receive prior cabozantinib or lenvatinib. The findings from cohort C suggest that sitravatinib’s activity is driven by inhibition of a similar scope of RTKs to cabozantinib and lenvatinib, and sitravatinib should not be further developed for patients whose cancer has progressed on cabozantinib or lenvatinib. At the time we developed SNAPI, the core question behind our study was whether sitravatinib could favorably modify the tumor microenvironment and restore sensitivity to ICI while also having independent anti-tumor activity. After our study was initiated, 2 phase III clinical trials evaluating this clinical scenario, CONTACT-03 and TiNivo-2, found that continuing an ICI while adding antiangiogenic targeted therapy did not improve survival in patients whose cancer has progressed on ICI. Since our ORR was 25% in cohorts A and B, we suspect sitravatinib was also ineffective at restoring sensitivity to ICI, and the clinical activity observed was due to the anti-tumor properities of sitravatinib alone. There is limited enthusiasm for further development of sitravatinib given the availability of similar agents in the post-ICI setting and introduction of belzutifan.

**Table:** 

Trial Information	
Disease	Clear cell renal cell carcinoma
Stage of disease/ treatment	Metastatic
Prior therapy	Immune checkpoint inhibitor (ICI)
Type of study	Phase II, single-arm, open label clinical trial
Primary endpoint	Objective response rate (ORR) per RECIST version 1.1 and disease control rate (DCR) at 24 weeks
Secondary endpoints	Overall survival (OS), progression-free survival (PFS), grade 3/4 adverse events per CTCAE version 5.0

## Additional details of endpoints or study design

The SNAPI clinical trial was a multicenter, single-arm, open label, phase II clinical trial performed at 3 institutions: The University of Texas MD Anderson Cancer Center (Houston, TX), Indiana University Melvin and Bren Simon Comprehensive Cancer Center (Indianapolis, IN), and the University of Michigan Rogel Cancer Center (Ann Arbor, MI). Patients were enrolled into 3 cohorts based upon prior treatment: cohort (1) progression on first-line (1L) nivolumab + ipilimumab, cohort, (2) progression on 1L pembrolizumab + axitinib or 2L anti-PD-1 therapy after receiving 1L VEGF-targeted monotherapy, and cohort (3) progression on 1L or 2L cabozantinib or lenvatinib ± everolimus either before, after, or in combination with anti-PD-1 ICI.

**Table:** 

Drug Information	
**Generic/working name**	Sitravatinib plus nivolumab
**Company name**	Bristol Myers Squibb
**Drug type**	Sitravatinib is a multitarget receptor tyrosine kinase (RTK) inhibitor. Nivolumab is a monoclonal antibody.
Drug class	Sitravatinib is a targeted therapy. Nivolumab is an ICI.
Dose	Sitravatinib 100 mg + nivolumab 480 mg
**Unit**	Milligrams (mg)
**Route**	Sitravatinib is oral. Nivolumab is intravenous (IV).
Schedule of administration	Sitravatinib is daily. Nivolumab is every 4 weeks.

**Table:** 

Patient Characteristics	
Number of patients, male	10 (71.4%)
Number of patients, female	4 (28.6%)
**Stage**	IV
Age: median (range)	55 years
Number of prior systemic therapies: median (range)	1 prior treatment: 6 (42.9%) 2 prior treatments: 8 (57.1%)
IMDC risk group	Favorable: 2 (14.3%) Intermediate: 11 (78.6%) Poor: 1 (7.1%)
Performance status: ECOG	0: 10 (71.4%) 1: 4 (28.6%) 2: 0 (0%) 3: 0 (0%) 4: 0 (0%)
Cancer types or histologic subtypes	Clear cell renal cell carcinoma: 14 (100%)

**Table:** 

Primary assessment method	
Number of patients screened	18
Number of patients enrolled	14
Number of patients evaluable for toxicity	14
Number of patients evaluated for efficacy	14
Evaluation method	RECIST 1.1
Response assessment, CR	0 (0%)
Response assessment, PR	2 (2%)
Response assessment, SD	7 (50%)
Response assessment, PD	5 (35.7%)
Median duration assessments, PFS	5.5 months (95% CI: 3.8—not reached)
Median duration assessments, OS	13.3 months (95% CI: 8.8—not reached)

## Assessment, analysis, and discussion

**Table:** 

Completion:	Study terminated prior to completion
Investigator’s assessment:	Level of activity did not meet planned end point

First-line treatment of metastatic clear cell renal cell carcinoma (ccRCC) consists of an immune checkpoint inhibitor (ICI) targeting PD-1 combined with either a CTLA-4 checkpoint inhibitor or antiangiogenic targeted therapy. While the introduction of ICI have meaningfully improved outcomes for patients with metastatic ccRCC, most will eventually progress and require subsequent lines of therapy. Contemporary second-line or later treatments produce limited benefit with median progression-free survival (PFS) of 10 months or less.^1^ Resistance to ICI can be mediated through multiple mechanisms, including an immune-suppressive tumor microenvironment (TME). Immune-suppressive cells, including myeloid-derived suppressor cells (MDSCs), immune-suppressive macrophages, regulatory T cells, and immature dendritic cells, express multiple receptor tyrosine kinases (RTKs) that contribute to an immune-suppressive TME when activated.^2^ Sitravatinib is an oral multi-kinase inhibitor targeting VEGFR, TAM, and MET. In pre-clinical studies, sitravatinib significantly reduced the number of immune-suppressive myeloid cells, increased CD4 + T-cells, and upregulated inflammatory genes at the transcriptome level.^3^ In translational studies from patients who received sitravatinib plus nivolumab after antiangiogenic targeted therapy, sitravatinib produced a proinflammatory state by decreasing immune-suppressive myeloid cells in the peripheral blood and increasing the T-cell to immune-suppressive myeloid cell ratio in the tumor.^4^ Thus, we hypothesized that sitravatinib plus nivolumab would revert an immune-suppressive TME to improve objective response rate (ORR) and survival in patients with metastatic ccRCC whose disease progressed on or after ICI.

SNAPI was an investigator-initiated, phase II, multicenter, single-arm trial where patients with progressive, metastatic ccRCC after 1-2 lines of treatment were enrolled into three cohorts: cohort (1) progression on 1L nivolumab + ipilimumab, cohort, (2) progression on 1L pembrolizumab + axitinib or 2L anti-PD-1 therapy after receiving 1L VEGF-targeted monotherapy, or cohort, (3) progression on 1L or 2L cabozantinib or lenvatinib ± everolimus either before, after, or in combination with anti-PD-1 ICI (NCT04904302). Patients were treated with sitravatinib 100 mg PO daily and nivolumab 480 mg IV every 4 weeks until disease progression or unacceptable toxicity. The co-primary endpoints of SNAPI were objective response rate (ORR), defined as complete response (CR) + partial response (PR), and disease control rate (DCR) at 24 weeks, defined as CR + PR + stable disease (SD). ORR was measured using RECIST version 1.1. Secondary endpoints included PFS, overall survival (OS), and incidence of ≥ grade 3 adverse events per the NCI Common Terminology Criteria for Adverse Events (CTCAE) version 5.0. The study was designed to enroll 88 patients with an interim analysis for futility in each cohort using a Bayesian Optimal Phase II (BOP2) design, but it was terminated early due to discontinuation of sitravatinib development.

**Figure 2.**
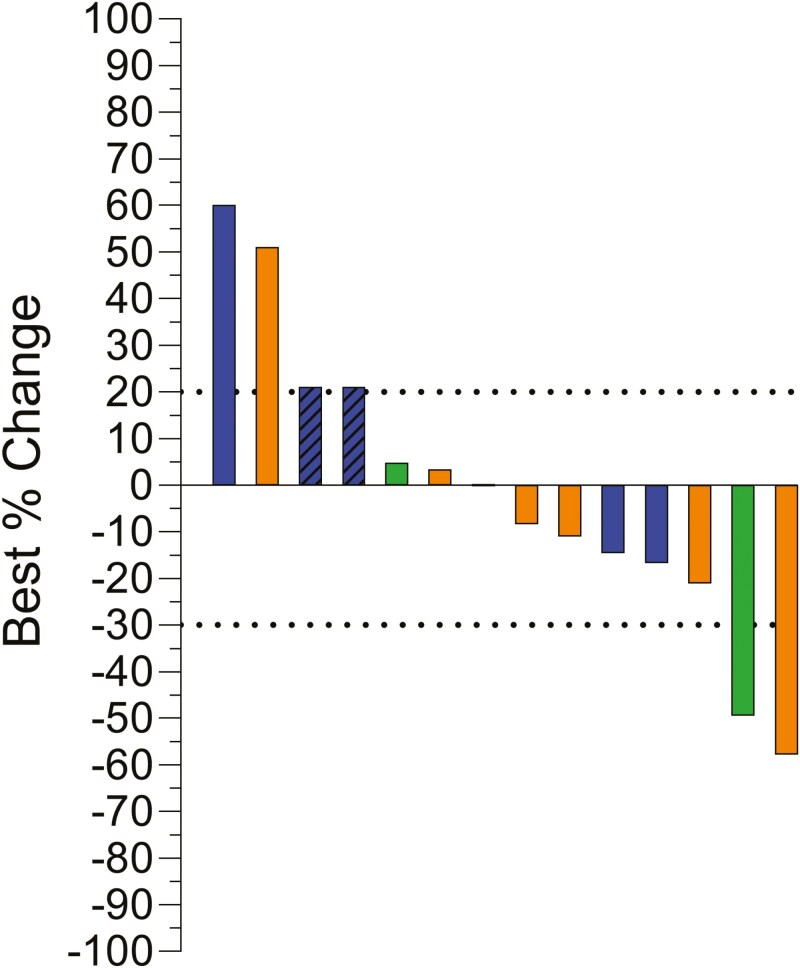
Waterfall plot depicting best percent change in the sum of tumor diameters per RECIST version 1.1. Hatches represent progressive disease due to either appearance of a new lesion or progression of non-measurable lesions. Green indicates cohort A, orange indicates cohort B, and blue indicates cohort C.

**Figure 3.**
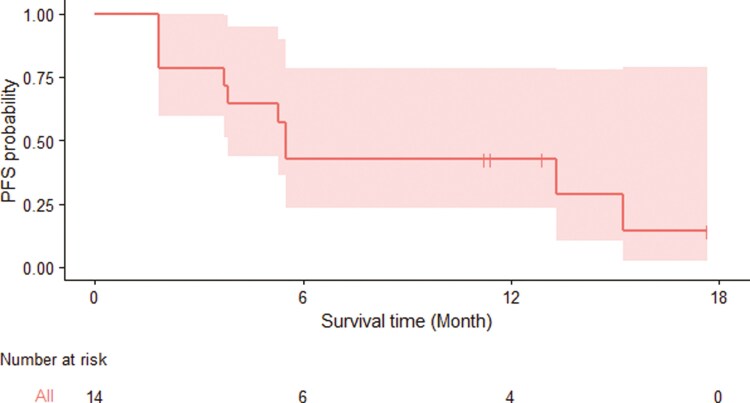
Kaplan-Meier curve depicting progression-free survival for the entire cohort. Red shading represents the 95% confidence interval.

Prior to termination, 14 patients were enrolled with 2 in cohort A, 6 in cohort B, and 6 in cohort C. Median age at enrollment was 50 years (range 40-75), and the majority were men (71.4%). IMDC risk score was favorable in 14.3%, intermediate in 78.6%, and poor in 7.1%. Two patients had sarcomatoid dedifferentiation. Sitravatinib plus nivolumab was given as second-line treatment in 42.9% of patients and third-line in 57.1%. In the overall cohort, the ORR at 24 weeks was 14.3% and the DCR at 24 weeks was 35.7% (Figures 1 and 2). The 2 patients who experienced a PR were pre-treated with nivolumab + ipilimumab and pembrolizumab + axitinib. In cohort A + B, the ORR was 25% and the DCR at 24 weeks was 62.5%. In contrast, the ORR was 0% and the DCR at 24 weeks was 0% (Figure 1) in cohort C. Median PFS was 5.5 months [95% CI 3.8—not reached (NR), Figure 3], and median OS was 13.3 months (95% CI 8.77—NR). 42.9% of patients experienced a grade 3-4 adverse event (AE) and 14.3% experienced an immune-mediated AE (**Table 1**). Dose interruptions were common (64.3%), and half of patients required a reduction in the dose of sitravatinib.

**Table 1:** Adverse events for patients who received sitravatinib plus nivolumab.

	Overall cohort (n = 14)
Any grade adverse events—no. (%)	14 (100%)
Grade 3/4 adverse events—no. (%)	6 (42.9)
Serious adverse events—no. (%)	7 (50%)
Immune related adverse events—no. (%)	
Any grade	2 (14.3%)
Grade 3/4	0 (0%)
Dose interruptions	9 (64.3%)
Dose reductions to sitravatinib	7 (50%)

Patients with metastatic ccRCC who progress on first-line ICI combinations have limited treatment options, and in our prior investigator-initiated trial, sitravatinib, a multi-kinase inhibitor of VEGFR, TAM, and MET, has favorably modified the TME by reducing immune-suppresive myeloid cells in the peripheral blood and increasing the T-cell to immune-suppresive myeloid cell ratio in the tumor.^4^ In this phase II trial, we investigated whether sitravatinib would improve clinical outcomes by having its own anti-tumor activity and restoring sensitivity to nivolumab in patients whose cancer has progressed on ICI. While the sample size was limited due to early study termination, sitravatinib plus nivolumab demonstrated a manageable safety profile and produced modest clinical benefit in patients who had not previously received cabozantinib or lenvatinib. Cabozantinib and lenvatinib target a similar spectrum of RTKs compared to sitravatinib, so after early termination, we chose to report findings in the overall cohort and in cohorts A/B (no prior cabozantinib or lenvatinib) versus cohort C. Sitravatinib did not have any clinical activity after progression on cabozantinib or lenvatinib as the ORR and DCR at 24 weeks were 0%. In contrast, we observed clinical activity in cohorts A/B where patients had only received ICI + VEGFR targeted therapy, such as axitinib.

When SNAPI was designed, it was unknown whether antiangiogenic targeted therapy could restore sensitivity to ICI through modulation of the TME. In the interim, 2 phase III clinical trials evaluating this clinical scenario, CONTACT-03 and TiNivo-2, found that continuing an ICI while adding antiangiogenic targeted therapy did not improve survival in patients whose cancer has progressed on ICI.^5,6^ While a randomized phase II trial would be needed to definitively know if sitravatinib uniquely modifies the TME, the ORR of 25% in cohorts A plus B does not compare favorably to the control arms of the aforementioned studies. The clinical development of sitravatinib was discontinued, and it is unclear how much enthusiasm would remain for further development of a multi-kinase targeted therapy given the availability of similar agents in the post-ICI setting and introduction of belzutifan. In conclusion, sitravatinib plus nivolumab demonstrated a manageable safety profile and produced modest clinical benefit in patients who had not previously received cabozantinib or lenvatinib, yet our sample size was limited due to the early termination of the SNAPI trial.

## Supplementary Material

oyaf053_suppl_Supplementary_Figure_S1

## Data Availability

The data underlying this article will be shared on reasonable request to the corresponding author.
