# Supplementary figures and images for: A phase II trial of sitravatinib + nivolumab after progression on immune checkpoint inhibitor in patients with metastatic clear cell RCC
Source: Oncologist. 2025 Apr 11;30(4):oyaf053. doi: 10.1093/oncolo/oyaf053 (PMC11986416; doi:10.1093/oncolo/oyaf053)

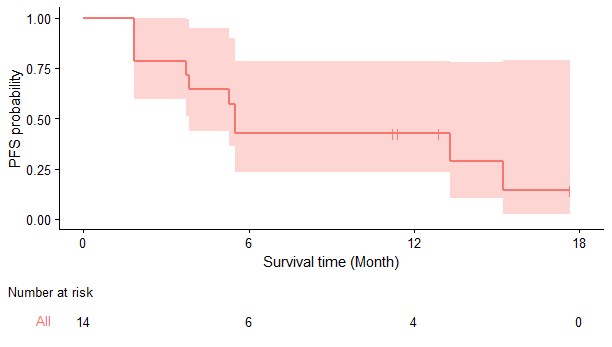

Supplement: oyaf053_suppl_Supplementary_Figure_S1 [file oyaf053_suppl_supplementary_figure_s1.jpeg]
